# Note for Male Smokers: The Healthy Eating Index Associated With the Risk of Subclinical Myocardial Injury

**DOI:** 10.1002/clc.70330

**Published:** 2026-04-27

**Authors:** Zhi‐jin Li, Bin‐gui Li

**Affiliations:** ^1^ Electrocardiogram Room Longyan First Affiliated Hospital of Fujian Medical University Longyan Fujian China; ^2^ Electrocardiogram and Electroencephalogram Room Laibin People's Hospital Laibin Guangxi China

**Keywords:** healthy eating index, NHANES III, smoke, subclinical myocardial injury

## Abstract

**Objectives:**

To investigate the associations of the healthy eating index (HEI) and subclinical myocardial injury (SCMI) in different smoking statuses (current smokers, former smokers, and never smokers) based on the National Health and Nutrition Examination Survey (NHANES) III.

**Methods:**

SCMI was defined as a cardiac infarction injury score (CIIS) ≥ 10. The HEI was compared between individuals with and without SCMI across different smoking status categories. Logistic regression analysis was used to investigate the association between HEI and SCMI stratified by smoking status, as well as in subgroups defined by sex and smoking status. Restricted cubic spline (RCS) modeling was applied to assess the nonlinear relationship between HEI and SCMI risk among male participants. Logistic regression‐based classification was further employed to explore the contribution of individual HEI components to SCMI risk in male smokers.

**Results:**

A total of 7256 participants (without SCMI group: 5389; SCMI group: 1867) were included. After stratifying by smoking status, a significant difference in HEI was found between the two groups among smokers. Multivariable logistic regression analysis revealed that HEI was inversely associated with the odds of SCMI in smokers. This association was particularly pronounced in male smokers. RCS analysis revealed a negative linear association between HEI and SCMI risk in male smokers. Diet variety and saturated fats were major contributors to this relationship.

**Conclusion:**

This study demonstrated that in male smokers, HEI scores negatively correlated with SCMI risk, with diet variety and saturated fats being major contributors in the late 1980s to early 1990s.

## Graphical abstract

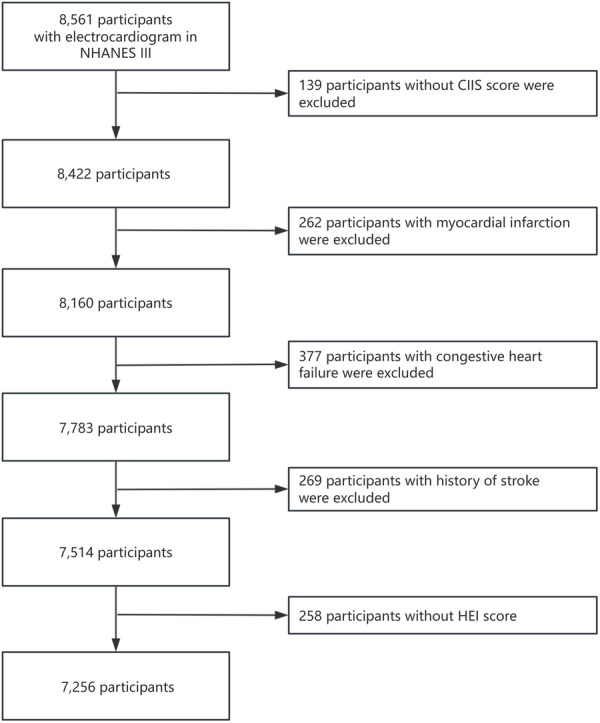

## 1 Introduction

The relationship between dietary quality and the risk of chronic diseases has become a key focus in public health research. The role of nutrition and diet in reducing the risk of chronic diseases, such as cardiovascular disease [1], diabetes mellitus [2], and certain forms of cancer [3], has been well documented. The Healthy Eating Index (HEI) is a composite measure of diet quality originally developed to assess conformance with the Dietary Guidelines for Americans. The version available in NHANES III comprises 10 components: 5 food‐group components (grains, vegetables, fruits, milk, and meat), 4 nutrient‐based components (total fat, saturated fat, cholesterol, and sodium), and a dietary variety component. The total score ranges from 0 to 100, with a higher score indicating better dietary quality [4]. Epidemiological evidence has shown that optimizing the HEI is significantly negatively correlated with the incidence of cardiovascular diseases [5]. The underlying mechanisms of this protective effect involve multiple pathways: Dietary patterns rich in antioxidants can scavenge reactive oxygen species (ROS) and inhibit low‐density lipoprotein (LDL) oxidation, thereby mitigating oxidative stress‐induced damage to the vascular endothelium [6, 7]. Concurrently, dietary patterns abundant in ω‐3 fatty acids and dietary fiber can downregulate the expression of pro‐inflammatory factors, leading to improved endothelial function [8]. These antioxidant and anti‐inflammatory pathways constitute the biological basis for the prevention of early myocardial injury by healthy dietary patterns.

It is noteworthy that subclinical myocardial injury (SCMI) refers to early heart damage without the clear manifestations of coronary heart disease (CHD); it is characterized by elevated high‐sensitivity cardiac troponin (hs‐cTn) levels [9]. It can be identified through multiple complementary approaches, including elevated hs‐cTn concentrations and electrocardiographic (ECG) scoring systems such as the Cardiac Infarction Injury Score (CIIS) [10]. SCMI is associated with an increased risk of cardiovascular events [11, 12], and can serve as an early warning for vascular events. The mechanism of its occurrence is closely related to oxidative stress and endothelial dysfunction caused by long‐term dietary patterns [13, 14].

However, among smokers, the association between dietary quality and SCMI may be fundamentally altered by the pathological effects of tobacco exposure. Smoking promotes the occurrence of SCMI through multiple independent and synergistic pathophysiological mechanisms. First, tobacco smoke contains a large number of free radicals and pro‑oxidants, which, upon entering the circulation, can directly reduce the bioavailability of endothelial nitric oxide (NO), leading to endothelial dysfunction [15]. Second, smoking‑induced oxidative stress, via activation of NADPH oxidase and the mitochondrial respiratory chain, continuously generates superoxide anions. These anions react with NO to form highly cytotoxic peroxynitrite, further aggravating endothelial injury and promoting the progression of atherosclerosis [16]. Third, smoking upregulates the expression of adhesion molecules and pro‑inflammatory factors through a systemic inflammatory response, promoting the adhesion of macrophages and platelets to the endothelium and creating a pro‑thrombotic and pro‑inflammatory microenvironment [17]. Notably, smoking also significantly depletes endogenous antioxidants such as vitamin C, vitamin E, β‑carotene, and glutathione, thereby increasing smokers’ demand for dietary‑derived antioxidants while simultaneously reducing their bioavailability [18]. This implies that even with a high‑quality diet, the oxidative damage induced by smoking may exceed the protective capacity provided by dietary antioxidants, thereby attenuating the cardioprotective effect of a healthy diet. Consequently, the impact of dietary quality on SCMI may differ substantially depending on smoking status.

Currently, there is a lack of systematic research on the dose‐response relationship between dietary quality scores and SCMI in different smoking status groups. Existing evidence mainly focuses on non‐smoking populations or uses cross‐sectional designs [19], making it difficult to differentiate between the protective effects of diet and the interactions with smoking‐related damage. This study, utilizing a cross‐sectional study based on data from the National Health and Nutrition Examination Survey (NHANES), aims to explore the relationship between HEI scores and SCMI events in smokers. Although this study also employed a cross‐sectional design, it differs from previous research in the following aspects: firstly, we systematically evaluated the association between the HEI and SCMI within a framework stratified by smoking status; secondly, the nonlinear dose‐response relationship between dietary quality and disease risk was untangled by constructing restricted cubic spline models, moving beyond reliance on simple linear assumptions; thirdly, the use of large‐sample, nationally representative data from NHANES enhances the generalizability of the findings.

## 2 Materials and Methods

### 2.1 Data Source and Study Population

The NHANES is a research program designed to assess the health and nutritional status of both adults and children in the United States. Initiated in the early 1960s, NHANES has conducted a series of surveys targeting various populations and health topics, utilizing interviews and physical examinations. The database has been approved by the National Center for Health Statistics Research Ethics Review Board, and all participants provided written informed consent. It should be noted that the CIIS is only available in NHANES III, which is why other survey years cycle were not selected.

**Figure 1:**
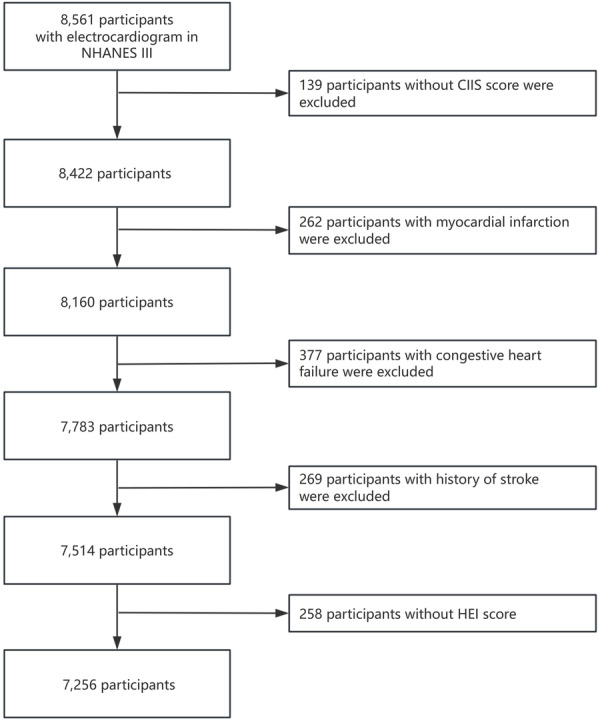
Flow chart of the study population.

The NHANES III database, which covers surveys conducted from 1988 to 1994, differs from the later 2‐year cycles of NHANES in certain aspects. For this retrospective observational study, we utilized electrocardiogram (ECG) data from individuals aged 40 and older within NHANES III. We collected records from participants who had ECG data available (*N *= 8561). Individuals without CIIS scores (*n* = 139), those with a history of myocardial infarction (*n *= 262), congestive heart failure (*n* = 377), stroke (*n *= 269), and those without HEI scores (*n* = 258) were excluded. In total, 7256 participants were included in the final analysis. The process for participant selection is summarized in Figure 1.

### 2.2 SCMI Assessment

In the mobile centers, professional technicians used the Marquette MAC 12 electrocardiograph (Marquette Medical Systems, Milwaukee, WI, USA) to conduct electrocardiogram (ECG) measurements, obtaining participants’ resting 12‐lead ECGs. The ECGs were processed by the Epidemiological Cardiovascular Research Center (EPICARE Center, Wake Forest School of Medicine, Winston‐Salem, North Carolina, USA), which extracted various important indicators from the ECG data. We directly downloaded the CIIS from the NHANES III official website and used these scores to diagnose SCMI. The CIIS risk assessment system is an electrocardiographic grading system developed based on static 12‐lead ECGs to identify ischemic SCMI. It calculates the final score based on the sum of eight binary features (Largest Q:R amplitude ratio, Q:R amplitude ratio, S amplitude (μV), T amplitude (negative phase) (μV), T amplitude (positive phase) (μV), Largest Q duration (msec), positive amplitude (μV), and T amplitude (negative phase) (μV)), three ternary (R amplitude, Q duration (msec), and T amplitude (positive phase) (μV)) and four continuous features (T amplitude (positive phase) (μV), R amplitude (μV), T amplitude (negative phase) (μV), and Q duration (msec)) via different assignments and calculation weights, thus assessing the severity of ischemic SCMI [10]. It is important to note that, to avoid using decimals, the CIIS scores in NHANES III were multiplied by a factor of 10. Therefore, before analysis, we divided the obtained CIIS scores by 10 to obtain the original values. Following previous methods [11], we defined a CIIS score ≥10 as SCMI in this study. Notably, hs‐cTn assays were not available in NHANES III (conducted 1988–1994), which predated the clinical development of these biomarkers; therefore, a biomarker‐based definition of SCMI could not be applied in this dataset.

### 2.3 HEI Assessment

The HEI in this study was downloaded directly from the NHANES III official website (https://wwwn.cdc.gov/nchs/nhanes/nhanes3/datafiles.aspx). The following 10 dietary components were included in the index based on different aspects of a healthful diet: Components 1–5 measure the degree to which a person's diet conforms to the Food Guide Pyramid serving recommendations for the Grain, Vegetable, Fruit, Milk, and Meat Groups. Component 6 measures total fat consumption as a percentage of total food energy intake. Component 7 measures saturated fat consumption as a percentage of total food energy intake. Component 8 measures total cholesterol intake. Component 9 measures total sodium intake. Component 10 examines the amount of variety in a person's diet over a 1‐day period. Specific food rating scales and calculation methods were presented in the *supplementary documents*.

### 2.4 Covariates Assessment

In this study, the covariates we included are as follows: age (year), poverty income ratio (PIR), time of drank alcohol in past 1 year (days), cotinine (ng/mL), white blood cell count (1000 cells/µL), lymphocyte number (1000 cells/µL), hemoglobin (g/dL), red cell distribution width (%), platelet count, serum C‐reactive protein (mg/dL), serum uric acid (umol/L), serum glucose (mmol/L), serum blood urea nitrogen (mmol/L), serum albumin (g/L), serum cholesterol (mmol/L), serum triglycerides (mmol/L), serum LDL cholesterol (mmol/L), serum HDL cholesterol (mmol/L), sex (male vs. female), race (White vs. Black vs. American), education level (below high school vs. high school vs. above high school), marital status (married vs. other), obesity (no vs. yes), smoking (cotinine, no vs. yes), smoker (questionnaire, never vs. former vs. now), arthritis (no vs yes), asthma (no vs. yes), chronic bronchitis (no vs. yes), thyroid disease (no vs. yes), diabetes (no vs. yes), hypertension (no vs. yes), heart attack (no vs. yes), osteoporosis (no vs. yes), metabolic syndrome (no vs. yes).

Hypertension was diagnosed according to the following criteria: systolic blood pressure (SBP) ≥ 140 mmHg and/or diastolic blood pressure (DBP) ≥ 90 mmHg, or a prior diagnosis of hypertension with current use of antihypertensive medication [20, 21]. Diabetes was defined as self‐reported physician‐diagnosed diabetes, use of insulin or oral hypoglycemic agents, fasting blood glucose >126 mg/dL, glycated hemoglobin (HbA1c) ≥ 6.5%, or an oral glucose tolerance test result ≥200 mg/dL [22]. Obesity was defined as a body mass index (BMI) ≥ 30 kg/m². Conditions such as arthritis, asthma, chronic bronchitis, thyroid disease, heart attack, osteoporosis, and metabolic syndrome were all based on self‐reported physician diagnoses. It is important to note that smoking status was determined using two classification methods. (1) Based on participants’ cotinine levels, smoking status was classified as follows: cotinine levels >15 ng/mL were considered as smoking, and ≤15 ng/mL were considered as non‐smoking [23]. This classification was denoted as smoking (cotinine) in the study. (2) Smoking status was categorized as never, former, or current smoker using two questions: lifetime consumption of ≥100 cigarettes and current smoking status. “Never” smokers answered “no” to the first question, “former” smokers answered “yes” then “no”, and “current” smokers answered “yes” to both [24]. This classification was denoted as smoker (questionnaire) in the study.

### 2.5 Statistical Analysis

Participants were divided into two groups: the SCMI group and the without SCMI group. Continuous variables between the two groups were compared using the Mann‐Whitney U test due to non‐normality of the data, and results were presented as medians with interquartile ranges [25%, 75%]. Categorical variables were compared using the chi‐square test and expressed as counts and percentages (%). Multivariate logistic regression analysis was conducted to explore the relationship between HEI and SCMI in different smoking status groups, adjusting for various confounding factors in different models based on theoretical relevance. The crude model was without adjustment. Model 1 was adjusted for age, sex, race, education level, marital status, PIR; Model 2 was adjusted for obesity, arthritis, chronic bronchitis, diabetes, hypertension, heart attack; and Model 3 was adjusted for white blood cell count, hemoglobin, red cell distribution width, serum C‐reactive protein, serum uric acid, serum glucose, serum blood urea nitrogen, serum albumin, serum cholesterol, serum triglycerides, serum HDL cholesterol.

Additionally, we used logistic regression analysis to explore the relationship between HEI and SCMI in different sexes combined with different smoking status, adjusting for demographic characteristics (age, race, education level, marital status, PIR). The results showed a significant association between HEI and SCMI in the male smoking group. Therefore, subsequent research focused on the male smoking population. Restricted cubic spline (RCS) analysis was performed to investigate the relationship between HEI and SCMI in this group, and trend regression analysis was used to explore the trend of their relationship. Finally, we applied logistic regression‐based classification to rank the importance of different HEI patterns, thereby assessing the contribution of various dietary patterns to SCMI.

## 3 Results

### 3.1 Study Population Characteristics

The median age of the included 7256 participants was 59 [46, 68], and the median HEI was 64.900 [54.900, 74.600]. Significant differences between the SCMI group and the without SCMI group were observed in age (64.000 [52.000, 73.000] vs. 56.000 [46.000, 68.000]), PIR (2.174 [1.201, 3.551] vs. 2.338 [1.237, 3.799]), cotinine (0.343 [0.087, 134.000] vs. 0.264 [0.084, 23.500]), white blood cell count (7.000 [5.800, 8.400] vs. 6.750 [5.600, 8.100]), hemoglobin (14.100 [13.150, 15.050] vs. 13.900 [13.000, 14.850]), red cell distribution width (13.200 [12.700, 13.900] vs. 13.000 [12.550, 13.600]), serum C‐reactive protein (0.210 [0.210, 0.600] vs. 0.210 [0.210, 0.440]), serum uric acid (333.100 [267.700, 392.600] vs. 315.200 [261.700, 374.700]), serum glucose (5.440 [4.940, 6.050] vs. 5.270 [4.880, 5.770]), serum blood urea nitrogen (5.360 [4.280, 6.780] vs. 5.000 [4.280, 6.430]), serum albumin (41.000 [39.000, 43.000] vs. 41.000 [39.000, 43.000]), serum cholesterol (5.610 [4.890, 6.410] vs. 5.530 [4.860, 6.230]), serum triglycerides (1.580 [1.080, 2.290] vs. 1.410 [1.000, 2.050]), and serum HDL cholesterol (1.220 [0.980, 1.530] vs. 1.270 [1.030, 1.550]) (all *p*< 0.05, Table 1). For categorical variables, including sex, race, education level, marital status, obesity, smoking (cotinine), smoker (questionnaire), arthritis, chronic bronchitis, diabetes, hypertension, heart attack, and metabolic syndrome, significant differences were also found between the two groups (*p *< 0.05, Table 2). Specifically, the proportions of males (50.723% vs. 46.150%), White (56.116% vs. 50.545%), individuals with education below high school level (29.348% vs. 26.106%), former smokers (32.512% vs. 31.379%), current smokers (26.406% vs. 21.303%), individuals with obesity (31.277% vs. 28.288%), individuals with detectable serum cotinine levels (32.282% vs. 25.820%), and individuals with arthritis (37.225% vs. 28.582%), chronic bronchitis (7.874% vs. 5.846%), diabetes (14.117% vs. 9.621%), hypertension (41.635% vs. 31.967%), heart attack (6.341% vs. 2.328%), and metabolic syndrome (42.130% vs. 32.271%) were all higher in the SCMI group compared to the without SCMI group. In contrast, the proportion of married individuals (67.787% vs. 63.192%) was higher in the Without SCMI group than in the SCMI group.

**Table 1:** Continuous characteristics among adults aged 40 years or older by SCMI.

Variables	Miss	Total (*n* = 7256)	Without SCMI (*n* = 5389)	SCMI (*n* = 1867)	*Z*	*p*
Age, (year)	0	59.000 [47.000, 70.000]	56.000 [46.000, 68.000]	64.000 [52.000, 73.000]	15.761	< 0.001
PIR	690	2.284 [1.227, 3.747]	2.338 [1.237, 3.799]	2.174 [1.201, 3.551]	2.524	0.012
HEI	0	64.900 [54.900, 74.600]	65.100 [55.100, 74.700]	64.400 [54.200, 74.300]	1.832	0.067
Time of drank alcohol in past 1 year, (days)	4386	0.000 [0.000, 0.000]	0.000 [0.000, 0.000]	0.000 [0.000, 0.000]	0.096	0.892
Cotinine, (ng/mL)	361	0.283 [0.085, 52.700]	0.264 [0.084, 23.500]	0.343 [0.087, 134.000]	−3.819	< 0.001
White blood cell count, (1000 cells/µL)	264	6.800 [5.600, 8.200]	6.750 [5.600, 8.100]	7.000 [5.800, 8.400]	−4.654	< 0.001
Lymphocyte number, (1000 cells/µL)	264	2.150 [1.750, 2.650]	2.150 [1.750, 2.650]	2.150 [1.750, 2.700]	−0.100	0.921
Hemoglobin, (g/dL)	265	13.950 [13.000, 14.900]	13.900 [13.000, 14.850]	14.100 [13.150, 15.050]	−4.332	< 0.001
Red cell distribution width, (%)	265	13.050 [12.600, 13.650]	13.000 [12.550, 13.600]	13.200 [12.700, 13.900]	−8.49	< 0.001
Platelet count	266	262.000 [221.500, 309.000]	263.000 [223.000, 308.500]	260.000 [216.000, 309.500]	1.875	0.061
Serum C‐reactive protein, (mg/dL)	320	0.210 [0.210, 0.500]	0.210 [0.210, 0.440]	0.210 [0.210, 0.600]	−4.405	< 0.001
Serum uric acid, (umol/L)	359	315.200 [261.700, 380.700]	315.200 [261.700, 374.700]	333.100 [267.700, 392.600]	−6.684	< 0.001
Serum glucose, (mmol/L)	361	5.270 [4.880, 5.880]	5.270 [4.880, 5.770]	5.440 [4.940, 6.050]	−6.497	< 0.001
Serum blood urea nitrogen, (mmol/L)	359	5.360 [4.280, 6.430]	5.000 [4.280, 6.430]	5.360 [4.280, 6.780]	−3.763	< 0.001
Serum albumin, (g/L)	359	41.000 [39.000, 43.000]	41.000 [39.000, 43.000]	41.000 [39.000, 43.000]	2.282	0.022
Serum cholesterol, (mmol/L)	272	5.530 [4.860, 6.280]	5.530 [4.860, 6.230]	5.610 [4.890, 6.410]	−3.122	0.002
Serum triglycerides, (mmol/L)	290	1.460 [1.020, 2.120]	1.410 [1.000, 2.050]	1.580 [1.080, 2.290]	−6.443	< 0.001
Serum LDL cholesterol, (mmol/L)	4181	3.490 [2.870, 4.110]	3.470 [2.870, 4.090]	3.540 [2.900, 4.190]	−1.795	0.073
Serum HDL cholesterol, (mmol/L)	335	1.240 [1.030, 1.550]	1.270 [1.030, 1.550]	1.220 [0.980, 1.530]	4.359	< 0.001

Abbreviations: LDL, low density lipoprotein; HDL, high density lipoprotein; HEI, healthy eating index; PIR, poverty income ratio; SCMI, subclinical myocardial injury.

**Table 2:** Categorical characteristics among adults aged 40 years or older by SCMI.

Variables	Total (*n* = 7256)	Without SCMI (*n* = 5389)	SCMI (*n* = 1867)	*χ* ^2^	*p*
Sex, *n*(%)				11.635	< 0.001
Male	3434 (47.326)	2487 (46.150)	947 (50.723)		
Female	3822 (52.674)	2902 (53.850)	920 (49.277)		
Race, *n*(%)				26.946	< 0.001
White	3622 (52.003)	2599 (50.545)	1023 (56.116)		
Black	1669 (23.963)	1229 (23.901)	440 (24.136)		
American	1674 (24.034)	1314 (25.554)	360 (19.748)		
Education level, *n*(%)				18.053	< 0.001
Below high school	1943 (26.941)	1398 (26.106)	545 (29.348)		
High school	3198 (44.343)	2351 (43.903)	847 (45.611)		
Above high school	2071 (28.716)	1606 (29.991)	465 (25.040)		
Marital status, *n*(%)				13.131	< 0.001
Married	4825 (66.607)	3649 (67.787)	1176 (63.192)		
Other	2419 (33.393)	1734 (32.213)	685 (36.808)		
Obesity, *n*(%)				6.002	0.014
No	5142 (70.944)	3861 (71.712)	1281 (68.723)		
Yes	2106 (29.056)	1523 (28.288)	583 (31.277)		
Smoking (cotinine), *n*(%)				27.610	< 0.001
No	5000 (72.516)	3798 (74.180)	1202 (67.718)		
Yes	1895 (27.484)	1322 (25.820)	573 (32.282)		
Smoker (questionnaire), *n*(%)				28.329	< 0.001
Never	3317 (45.714)	2550 (47.319)	767 (41.082)		
Former	2298 (31.670)	1691 (31.379)	607 (32.512)		
Now	1641 (22.616)	1148 (21.303)	493 (26.406)		
Arthritis, *n*(%)				48.596	< 0.001
Yes	2235 (30.806)	1540 (28.582)	695 (37.225)		
No	5020 (69.194)	3848 (71.418)	1172 (62.775)		
Asthma, *n*(%)				1.093	0.296
Yes	498 (6.864)	360 (6.682)	138 (7.392)		
No	6757 (93.136)	5028 (93.318)	1729 (92.608)		
Chronic bronchitis, *n*(%)				9.557	0.002
Yes	462 (6.368)	315 (5.846)	147 (7.874)		
No	6793 (93.632)	5073 (94.154)	1720 (92.126)		
Thyroid disease, *n*(%)				1.011	0.315
Yes	372 (5.128)	268 (4.975)	104 (5.570)		
No	6882 (94.872)	5119 (95.025)	1763 (94.430)		
Diabetes, *n*(%)				29.095	< 0.001
Yes	781 (10.777)	518 (9.621)	263 (14.117)		
No	6466 (89.223)	4866 (90.379)	1600 (85.883)		
Hypertension, *n*(%)				57.151	< 0.001
Yes	2490 (34.454)	1716 (31.967)	774 (41.635)		
No	4737 (65.546)	3652 (68.033)	1085 (58.365)		
Heart attack, *n*(%)				67.955	< 0.001
Yes	241 (3.361)	124 (2.328)	117 (6.341)		
No	6930 (96.639)	5202 (97.672)	1728 (93.659)		
Osteoporosis, *n*(%)				0.418	0.518
Yes	225 (3.111)	163 (3.033)	62 (3.335)		
No	7008 (96.889)	5211 (96.967)	1797 (96.665)		
Metabolic syndrome, *n*(%)				54.541	< 0.001
No	4368 (65.213)	3379 (67.729)	989 (57.870)		
Yes	2330 (34.787)	1610 (32.271)	720 (42.130)		

Abbreviation: SCMI, subclinical myocardial injury.

### 3.2 Comparison of HEI Among Participants by SCMI in Different Smoking Statuses

We compared HEI levels between the SCMI group and the without SCMI group in different smoking status subgroups. We found a significant difference between the SCMI and without SCMI groups in the smoker population (*p *< 0.05, Table 3), but there was no difference between the two groups in non‐smokers (*p *> 0.05, Table 3). This result preliminarily suggests that a healthy diet may be associated with SCMI, particularly in the smoking population.

**Table 3:** HEI among adults aged 40 years or older by SCMI in different smoking status.

Group	Total (*n* = 5000)	Without SCMI (*n* = 5389)		SCMI (*n* = 1867)		*z*	*p*
Smoking (cotinine)							
No	67.000 [57.200, 76.400]	66.900 [57.100, 76.300]		67.500 [57.400, 77.100]		1.024	0.306
Yes	58.500 [50.100, 67.900]	59.200 [50.400, 68.100]		57.200 [49.400, 67.600]		2.148	0.032
Smoker (questionnaire)							
Never	67.200 [57.300, 76.600]	67.400 [57.400, 76.800]		66.800 [56.900, 75.600]		0.965	0.334
Former	65.800 [55.700, 75.000]	65.300 [55.500, 74.600]		67.100 [56.700, 76.500]		−2.095	0.036
Now	58.500 [49.800, 67.900]	59.600 [50.200, 68.600]		56.700 [48.900, 67.200]		2.839	0.005

Abbreviation: SCMI, subclinical myocardial injury.

### 3.3 Association of HEI and SCMI in Different Smoking Status

We further explored the relationship between HEI and SCMI in different smoking status groups. The logistic regression analysis results indicated that in the smoking groups defined by cotinine levels, HEI was inversely associated with SCMI (OR 95% CI: 0.991 [0.984, 0.999], *p* < 0.05), and this association remained significant in both Model 1 (OR 95% CI: 0.989 [0.981, 0.997], *p* < 0.05) and Model 2 (OR 95% CI: 0.990 [0.983, 0.998], *p* < 0.05). The p value for their association was close to 0.05 in Model 3. In the “former” group defined by the questionnaire, HEI was positively associated with SCMI in the crude model and Model 3 (OR 95% CI: 1.012 [1.005, 1.019], *p* < 0.05). It followed that their association may be affected by the confounding factors. In the “now” group, HEI was significantly associated with the occurrence of SCMI (OR 95% CI: 0.988 [0.980,0.996], *p* < 0.05), and the relationship was not influenced by other confounding factors (Model 1 OR 95% CI: 0.988 [0.979, 0.997]; Model 2 OR 95% CI: 0.987 [0.979,0.995]; Model 3 OR 95% CI: 0.989 [0.980, 0.997], all *p* < 0.05, Table S1), which suggesting their stable association in this subgroup.

**Figure 2:**
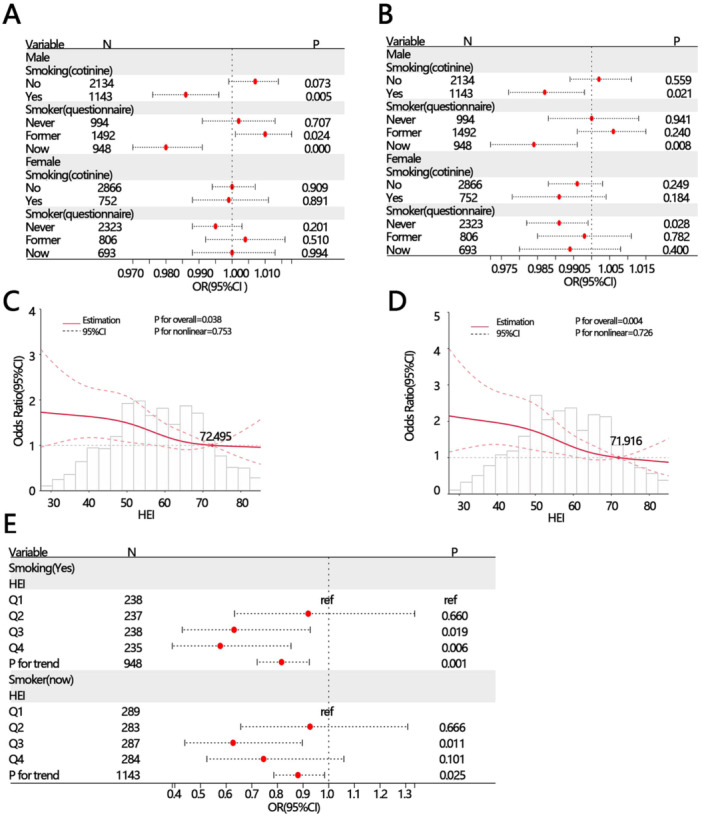
Relationship between HEI and SCMI stratified by smoking status in different sexes. (A) Logistic regression analysis without adjustment. (B) Logistic regression analysis was conducted, adjusting for age, race, education level, marital status, and PIR. (C) RCS analysis between HEI and SCMI in the smoking (yes) group of males. (D) RCS analysis between HEI and SCMI in the smoker (now) group of males. (E) Trend regression analysis between HEI and SCMI in males. CI, confidence; HEI, healthy eating index; OR, odds ratio; PIR, poverty income ratio; RCS, restricted cubic spline; SCMI, subclinical myocardial injury.

Additionally, sex may be associated with smoking status, as male smokers are generally more prevalent. Therefore, based on different sexes, we further examined the correlation between HEI and SCMI, stratified by smoking status. The results showed that in the male smoking (yes) and smoker (now) groups, HEI was significantly and steadily associated with SCMI (Figure 2A,B). In contrast, in females, no association between HEI and SCMI was observed. Subsequently, we conducted RCS analysis to explore the relationship between HEI and SCMI in the male smoking population. The RCS results showed a significant linear correlation between HEI and SCMI in the male group (Figure 2C,D). Furthermore, trend regression analysis revealed a trend where the risk of SCMI decreased as HEI increased (*p* for trend <0.05, Figure 2E).

### 3.4 Association of HEI Components and SCMI

**Figure 3:**
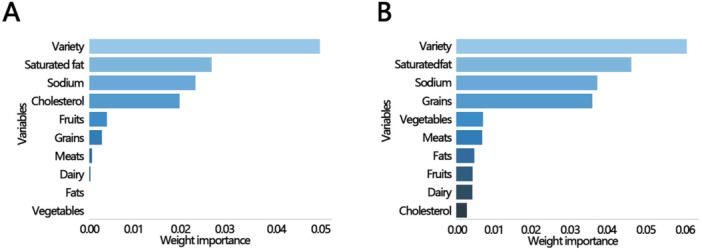
The importance ranking bar chart of the HEI components in (A) the smoking (yes) group of males and (B) the smoker (now) group of males. HEI, healthy eating index.

Based on the above findings, the study indicates a negative linear relationship between HEI and the risk of SCMI in the male smoking population. Therefore, we further explored the contribution of different HEI components to SCMI. Using logistic regression‐based classification and ranking, we performed importance ranking for the components of HEI. The results showed that in both smoking status groups, the variety of diet had the greatest contribution to the risk of SCMI, followed by saturated fatty acids (Figure 3A,B).

## 4 Discussion

This study identified a significant association between the HEI and SCMI, particularly within the smoking population based on NHANES III in the late 1980s to early 1990s. Additionally, higher HEI scores correlated with lower risk SCMI. The negative linear relationship between HEI and SCMI risk was especially pronounced among male smokers. In this relationship, dietary variety from the HEI index was the primary contributor to SCMI risk, followed by saturated fatty acids.

The study found that smoking status affects the correlation between healthy diet and SCMI risk. Smoking is a well‐established risk factor for cardiovascular disease. Polycyclic aromatic hydrocarbons generated during tobacco combustion can directly induce mitochondrial DNA damage in cardiomyocytes. In addition, nicotine promotes the polarization of macrophages toward a pro‐inflammatory phenotype by activating the α7 nicotinic acetylcholine receptor (α7nAChR), thereby triggering systemic inflammation, accelerating atherosclerosis, and impairing endothelial function [25]. As such, smoking directly increases the risk of cardiovascular events [25].

Therefore, smoking not only elevates the risk of myocardial injury through its own deleterious effects but may also alter the cardioprotective impact of a healthy diet. A healthy dietary pattern—particularly one rich in antioxidants, dietary fiber, and unsaturated fatty acids—is believed to attenuate oxidative stress, enhance endothelial function, and slow the progression of atherosclerosis [26]. However, in individuals who smoke, the oxidative stress and inflammatory responses induced by smoking may counteract these protective dietary effects, potentially diminishing the preventive benefits of a healthy diet against myocardial injury. Aldehydes such as acrolein, present in tobacco smoke, have been shown to specifically inhibit mitochondrial complex I activity in cardiomyocytes. Nevertheless, sufficient dietary intake of coenzyme Q10 and alpha‐lipoic acid may reverse this inhibitory effect [27, 28].

We also found that among male smokers, higher HEI scores were inversely associated with the risk of SCMI. This may be attributable to the higher prevalence of smoking among men, making them more susceptible to the adverse cardiovascular effects of tobacco use. One speculative explanation is that male smokers may be more likely than female smokers to have elevated blood cadmium levels [29]. In experimental studies, cadmium has been shown to promote the release of pro‐inflammatory cytokines such as TNF‐α and IL‐6 via NF‐κB signaling, potentially exacerbating myocardial inflammation [30, 31]. Additionally, cadmium may disrupt calcium regulatory proteins in cardiomyocytes, potentially contributing to contractile dysfunction [32]. However, in the absence of measured blood cadmium data in NHANES III, this pathway remains hypothetical, and the observed association between HEI and SCMI. A higher HEI score reflects a dietary pattern more consistent with established health recommendations [4], and is indicative of greater antioxidant capacity and enhanced immune function [33]. Additionally, dietary variety contributed most strongly to the protective association between HEI and SCMI risk among male smokers. From a biological standpoint, dietary variety ensures the simultaneous intake of a broader spectrum of bioactive compounds, including polyphenols, carotenoids, flavonoids, vitamins C and E, and omega‐3 fatty acids, derived from diverse food sources [34]. These phytochemicals exert synergistic antioxidant effects that exceed the sum of their individual contributions: for example, vitamin C regenerates oxidized vitamin E in cell membranes, while the pro‐oxidant or antioxidant activity of β‐carotene and lycopene depends on their interaction with co‐antioxidant molecules [35]. This synergistic antioxidant network may be particularly relevant in smokers, whose endogenous antioxidant reserves are substantially depleted by tobacco‐induced oxidative stress, making them more dependent on a diverse array of exogenous dietary antioxidants to counteract the multifaceted oxidative damage to the myocardium. In contrast, a diet lacking variety may fail to provide the full complement of micronutrients needed to sustain this cooperative antioxidant defense. From a behavioral perspective, greater dietary variety often serves as a marker of overall dietary quality and health consciousness, reflecting more balanced food choices across multiple food groups and a lower reliance on energy‐dense, nutrient‐poor foods that are commonly consumed by smokers [36]. Thus, the prominent role of dietary variety in our findings likely reflects both the biological synergy of diverse phytochemical intake and its function as an integrative behavioral indicator of a health‐promoting dietary pattern.

The counterintuitive finding that higher HEI was associated with increased SCMI risk among former smokers, in contrast to the protective effect observed in current smokers, is most plausibly explained by the “sick quitter” effect and reverse causation. The “sick quitter” hypothesis posits that individuals often quit smoking in response to emerging health problems rather than as a purely preventive behavior, and may subsequently adopt healthier dietary habits as part of broader lifestyle modification [37]. In our cross‐sectional design, this creates temporal ambiguity: the improved diet quality observed at assessment may reflect a behavioral response to pre‐existing subclinical cardiac damage rather than a causal contributor to it. Supporting this interpretation, a recent NHANES‐based study demonstrated that former smokers had significantly higher HEI scores than current smokers, with dietary quality improving rapidly after cessation [38]. Furthermore, former smokers have been shown to carry a greater burden of prevalent cardiovascular disease and cardiometabolic risk factors compared to both current and never smokers, consistent with the “sick quitter” profile [39]. Thus, the observed association among former smokers likely reflects reverse causation. Specifically, individuals with pre‐existing SCMI may have adopted healthier diets after quitting, rather than healthy eating exerting a deleterious effect. Prospective studies are needed to disentangle the temporal relationship between dietary improvement, smoking cessation, and subclinical cardiac outcomes.

Several methodological considerations regarding the use of CIIS to define SCMI merit discussion. While hs‐cTn has become the preferred biomarker for myocardial injury detection per the Fourth Universal Definition of Myocardial Infarction [40], NHANES III was conducted between 1988 and 1994, predating the clinical availability of hs‐cTn assays. Although hs‐cTn has since been measured in stored samples from NHANES 1999–2004 [41], such data are unavailable for the NHANES III cohort. Importantly, the CIIS has been extensively validated as a reliable measure of SCMI. In its original derivation, the CIIS demonstrated a sensitivity of 85% and specificity of 95% for detecting myocardial infarction [10]. In a comparative study of 46,933 patients, the CIIS outperformed all other ECG classification systems in predicting cardiovascular mortality (HR = 1.39 per tertile increase, 95% CI 1.32–1.45) [42]. Its prognostic value has been confirmed in apparently healthy populations over 28 years of follow‐up [43], and in middle‐aged men where elevated CIIS predicted coronary heart disease death (relative risk 5.8, 95% CI 3.4–9.9) [44]. Moreover, using the same NHANES III dataset, CIIS ≥ 10 was independently associated with cardiovascular mortality (HR = 1.26, 95% CI 1.02–1.56) and all‐cause mortality (HR = 1.42, 95% CI 1.23–1.63) among individuals free of CVD [11].

We recognize that CIIS and hs‐cTn are not interchangeable—hs‐cTn indicates active cardiomyocyte necrosis, while CIIS reflects the cumulative electrical footprint of previous injury. Currently, there are no direct comparisons of these two methods in population‐based settings. Future research with both CIIS and hs‐cTn data collected simultaneously is needed to determine their agreement. Nonetheless, the consistent link between CIIS‐defined SCMI and adverse outcomes across multiple independent cohorts supports its validity in this analysis.

### 4.1 Clinical Significance and Limitations

To the best of our knowledge, this study is the first to reveal that HEI is significantly associated with the risk of SCMI in male smokers. The findings suggest that in the context of NHANES III (1988–1994), higher diet quality was associated with lower odds of SCMI among male smokers. These results may provide a basis for generating hypotheses regarding dietary interventions for smokers, though validation in contemporary prospective cohorts with modern biomarkers is needed before clinical recommendations can be made. Therefore, we plan to conduct a prospective study to explore the impact of different dietary patterns on SCMI and to investigate the underlying mechanisms through animal experiments.

However, this study still had several limitations. First, the cross‐sectional design limits our ability to establish a causal relationship between the HEI and the SCMI. Second, the NHANES III data were collected between 1988 and 1994, and modern dietary patterns, smoking habits, and exposure to environmental pollutants may have changed, limiting the timeliness of the findings. Third, since NHANES III surveyed U.S. populations, the results may be regionally or ethnically biased. Fourth, the HEI relies on 24‐h dietary recalls or food frequency questionnaires, which may be subject to recall bias or misreporting. Additionally, the construction of the diet index does not encompass all nutrients, potentially affecting the accuracy of the results. Fifth, due to the absence of blood heavy metal levels in the NHANES III database, we lack corresponding data to validate our hypothesis that male smokers are more susceptible to SCMI as a result of elevated blood cadmium levels and subsequent inflammation. Finally, although we adjusted for multiple confounding factors in our analysis, we could not fully control for other potential confounders, such as physical activity, occupation, alcohol intake patterns, or psychosocial factors, which could introduce bias into the results.

## 5 Conclusion

In conclusion, this study found that in male smokers, the HEI index had a negative linear relationship with the risk of SCMI. Diet variety and saturated fatty acids have a greater contribution to SCMI in this population. These findings may provide hypotheses for future prospective studies and warrant validation in contemporary populations. If confirmed, dietary counseling emphasizing dietary diversity and saturated fat reduction could potentially complement conventional smoking cessation strategies for cardiovascular risk management. Future prospective studies using contemporary cohort data and modern biomarkers are warranted to validate these findings and further refine targeted nutritional interventions for smoking populations.

## Author Contributions

Z.J.L. contributed to the conception and design. Z.J.L. and B.G.L. contributed to the collection and assembly of data. Z.J.L. and B.G.L. analyzed and interpreted the data. All authors wrote and approved the final manuscript.

## Ethics Statement

The Ethics Committee of Longyan First Hospital deemed that this research is based on open‐source data, so the need for ethics approval was waived.

## Conflicts of Interest

The authors declare no conflicts of interest.

## Supporting information

Supporting File

## Data Availability

The data that support the findings of this study are available from the corresponding author upon reasonable request.
