# Supplementary material for: Note for Male Smokers: The Healthy Eating Index Associated With the Risk of Subclinical Myocardial Injury
Source: Clin Cardiol. 2026 Apr 27;49(5):e70330. doi: 10.1002/clc.70330 (PMC13112598; doi:10.1002/clc.70330)
Supplement: Supplementary file 1 — Supporting File [file CLC-49-e70330-s001.docx]

| **Table S1 Logistic regression analysis between HEI and SCMI in different smoking statuses** | | | | | | | | |
| --- | --- | --- | --- | --- | --- | --- | --- | --- |
|  | Crude model | | Model 1 |  | Model 2 |  | Model 3 |  |
| Group | OR[95%CI] | p | OR[95%CI] | p | OR[95%CI] | p | OR[95%CI] | p |
| Smoking(cotinine) | |  |  |  |  |  |  |  |
| No | 1.003[0.998,1.007] | 0.289 | 0.999[0.993,1.004] | 0.604 | 1.002[0.997,1.007] | 0.532 | 1.004[0.999,1.009] | 0.153 |
| Yes | 0.991[0.984,0.999] | 0.020 | 0.989[0.981,0.997] | 0.009 | 0.990[0.983,0.998] | 0.012 | 0.993[0.985,1.000] | 0.066 |
| Smoker(questionnaire) | | |  |  |  |  |  |  |
| Never | 0.997[0.991,1.003] | 0.306 | 0.993[0.987,1.000] | 0.062 | 0.996[0.989,1.002] | 0.166 | 0.997[0.990,1.003] | 0.287 |
| Former | 1.008[1.001,1.015] | 0.028 | 1.003[0.995,1.011] | 0.468 | 1.007[1.000,1.014] | 0.065 | 1.012[1.005,1.019] | 0.001 |
| Now | 0.988[0.980,0.996] | 0.004 | 0.988[0.979,0.997] | 0.010 | 0.987[0.979,0.995] | 0.002 | 0.989[0.980,0.997] | 0.010 |

Crude model: without adjustment.

Model 1: adjusting for age, sex, race, high school, marital status, PIR.

Model 2: adjusting for obesity, arthritis, chronic bronchitis, diabetes, hypertension, heart attack.

Model 3: adjusting for white blood cell count, hemoglobin, red cell distribution width, serum C-reactive protein, serum uric acid, serum glucose, serum blood urea nitrogen, serum albumin, serum cholesterol, serum triglycerides, serum HDL cholesterol.

Abbreviation: SCMI: subclinical myocardial injury, PIR: poverty income ratio, HEI: healthy eating index, HDL: high density lipoprotein, OR: odds ratio; CI: confidence interval.
